# An Iterated Tabu Search Approach for the Clique Partitioning Problem

**DOI:** 10.1155/2014/353101

**Published:** 2014-03-04

**Authors:** Gintaras Palubeckis, Armantas Ostreika, Arūnas Tomkevičius

**Affiliations:** Faculty of Informatics, Kaunas University of Technology, Studentu Street 50-408, 51368 Kaunas, Lithuania

## Abstract

Given an edge-weighted undirected graph with weights specifying dissimilarities between pairs of objects, represented by the vertices of the graph, the clique partitioning problem (CPP) is to partition the vertex set of the graph into mutually disjoint subsets such that the sum of the edge weights over all cliques induced by the subsets is as small as possible. We develop an iterated tabu search (ITS) algorithm for solving this problem. The proposed algorithm incorporates tabu search, local search, and solution perturbation procedures. We report computational results on CPP instances of size up to 2000 vertices. Performance comparisons of ITS against state-of-the-art methods from the literature demonstrate the competitiveness of our approach.

## 1. Introduction

Clique partitioning is an important combinatorial optimization problem with many real-life applications. It can be stated as follows. Suppose that there are *n* objects and, in addition, there is an *n* × *n* symmetric matrix, *D* = (*d*_*ij*_), whose entry *d*_*ij*_ represents the dissimilarity between objects *i* and *j*. These data can be modeled by considering a complete edge-weighted undirected graph with vertices corresponding to objects and edge weights given by the dissimilarity matrix *D*. Let this graph be denoted by *G* and its vertex set by *V*. The clique partitioning problem (CPP for short) is to partition the vertex set *V* into mutually disjoint subsets such that the sum of the edge weights over all cliques induced by the subsets is as small as possible. Henceforth, we will denote a feasible solution to the CPP as *P* = (*V*_1_,…, *V*_*m*_), where *m* ∈ [1,…, *n*], ⋃_*k*=1_^*m*^ *V*_*k*_ = *V*, and *V*_*k*_∩*V*_*l*_ = *∅* for each pair *k*, *l* ∈ {1,…, *m*}, *k* ≠ *l*. The set of all such solutions is denoted by Π. Mathematically, the clique partitioning problem can be expressed as

(1) $$\begin{array}{c} \underset{P\in \Pi }{\min}F\left(P\right)=\overset{m}{\underset{k=1}{\sum }}\;\underset{i,j\in V_{k},i<j}{\sum }d_{ij}. \end{array}$$

The CPP bears some resemblance to the maximally diverse grouping problem (MDGP) [1–3]. There are two main differences between the CPP and the MDGP. First, the latter assumes that the number of groups is fixed a priori. Meanwhile, in the case of CPP, the number of clusters is allowed to vary throughout solution process and is part of the output of algorithms designed for the CPP. Second, in the MDGP, the size of each group is either fixed or bounded from above and possibly from below. No constraints are imposed on the size of clusters in the formulation of the CPP. It follows from the above observations that nontrivial instances of (1) are defined by dissimilarity matrices with both positive and negative entries.

The clique partitioning problem is of interest in several contexts, one of them being combinatorial data analysis. In this context, the objects are characterized by a set of attributes. The values of an attribute are represented by a binary equivalence relation. Then the CPP can be interpreted as the problem of aggregation of binary relations. In this case, the entry *d*_*ij*_ of the matrix *D* represents the number of attributes on which objects *i* and *j* disagree minus the number of attributes on which they agree. More details on this approach to data analysis can be found, for example, in [4–7]. The applicability of the CPP has also been reported in a number of other settings such as assigning flights to airport gates [8, 9], modularity maximization in networks [10], genomics [11], clustering [12, 13], and group formation in cellular manufacturing [14, 15].

Given the practical importance of the problem, many methods, both exact and heuristic, have been proposed in the literature. One of the first exact algorithms for the CPP was developed by Grötschel and Wakabayashi [16]. Their algorithm is based on the cutting plane technique. It uses polyhedral results presented in the companion paper [17]. Several new classes of facet-defining inequalities of the clique partitioning polytope were introduced by Oosten et al. [14]. The usefulness of the inequalities was demonstrated by performing experiments on a set of CPP instances arising in flexible manufacturing. Branch-and-bound algorithms for the CPP were proposed in [18, 19]. Computational results reported in [18, 19] show that these algorithms perform significantly better than the cutting plane method of Grötschel and Wakabayashi. Mehrotra and Trick [20] developed a branch-and-price algorithm for solving the CPP. Actually, their approach is applicable to the formulation that captures both the CPP and the capacitated clustering problem. Sukegawa et al. [21] presented a problem size reduction technique based on the Lagrangian relaxation and the pegging test. Its validity has been verified through extensive numerical experiments. Recently, a branch-and-bound algorithm for the CPP was proposed by Jaehn and Pesch [22]. Their algorithm incorporates several useful features including improved constraint propagation techniques for fixing edges at the nodes of the search tree.

It is well known that the CPP given by (1) is NP-hard in its general form. Thus, CPP instances of larger size can be solved only using heuristic algorithms. Perhaps the most traditional way to approach a combinatorial optimization problem is to resort to local search (LS) techniques. LS algorithms for solving the CPP were developed by Régnier [23] and by Marcotorchino and Michaud [24]. A multistart LS procedure was considered by Guénoche [7]. The drawback of LS techniques is that they might get trapped into poor quality local optima. The other way to approach the problem is to use metaheuristic search methods. Simulated annealing and tabu search implementations for the CPP were proposed by de Amorim et al. [25]. It was found that they performed very favourably in comparison to Régnier's heuristic. Kochenberger et al. [11] presented an approach that relies on the idea of recasting the CPP into the form of a binary quadratic program. The program is solved using a tabu search method incorporating strategic oscillation. Dorndorf and Pesch [18] proposed an ejection chain algorithm for clique partitioning. The algorithm takes advantage of the variable depth local search strategy of Kernighan and Lin [26]. Charon and Hudry [27] offered an adaptation of the noising method to the CPP. They investigated a number of variations of this method, differing in the way of adding noise to the data. Computational experiments were carried out on graphs of order up to 500. Brusco and Köhn [6] developed two versions of the neighborhood search algorithm with different search intensification components. Specifically, the first version uses Régnier's LS procedure, whereas the second one uses the tabu search algorithm. Both versions of the method were shown to be superior to simulated annealing and tabu search implementations from [25].

The focus of this paper is on developing an iterated tabu search (ITS) algorithm for solving the CPP. The primary intention is to combine search intensification and diversification components in order to achieve better performance, compared with that achievable by the best methods in the literature. Our strategy is to test the algorithm on a suite of larger CPP instances than those considered in the previous studies. We report computational experience on problem instances whose size goes up to 2000 vertices. We compare our ITS technique against the heuristics of Brusco and Köhn [6], which are the most successful of the current algorithms for the clique partitioning problem.

It should be noted that various implementations of the iterated tabu search method have also been proposed for other optimization problems. Excellent results have been reported in a number of papers, including [28–34]. The tabu search metaheuristic used in ITS is a general-purpose optimization method, based on which specific algorithms for a variety of optimization problems have been developed. The origins of tabu search go back to the seminal work of Glover [35]. The basic concepts of the modern form of tabu search have been presented by Glover in [36].

The remainder of this paper is arranged as follows. In the next section, we present a detailed description of the ITS algorithm for the CPP. In Section 3, we report the results of computational experiments. Concluding remarks are given in the last section.

## 2. The Algorithm

In this section, we describe the components of our iterated tabu search algorithm for solving the clique partitioning problem. The essence of the algorithm is simple: improve an initial partition by repeatedly applying tabu search (TS) and solution perturbation procedures. Run duration of the first of them is controlled by imposing a limit on the number of iterations. Upon discovering an improving solution by TS, this solution is submitted to a local search procedure, which explores its neighborhood in an attempt to make further improvements. The implementation details of all these ingredients of the approach are explained next.

### 2.1. General Scheme

An important aspect of the clique partitioning problem is that the number of clusters is not fixed. Thus, not only the content of the clusters but also their number is varying throughout the search process. In this regard, it is convenient to have an empty cluster added to the partition vector *P*. We assume in the description of algorithms that *m* counts all clusters including the empty one. There are two possible scenarios in managing a partition when the number of clusters is changing. An easy case is when a vertex is ordered to move to the empty cluster in *P*. In this case, a new empty cluster is added to the current partition. More processing is required if the last vertex of some cluster, say *V*_*l*_, is relocated. In this case, *V*_*l*_ becomes empty. A possible strategy is to remove *V*_*l*_ from the partition *P* and renumber the remaining clusters. However, in our approach, renumbering may prove to be unduly time consuming. In particular, this operation implies the need for additional computations when updating tabu information. We have implemented another strategy, which rests on the idea of retaining all emptied clusters. We use a bit-vector of flags (*a*_1_,…, *a*_*m*_). Its components are set to 0 for all nonempty clusters as well as precisely one selected empty cluster. Let the latter be denoted by *V*_*e*_. The components of the vector corresponding to other empty clusters are equal to 1. Thus, in the situation outlined above, the cluster *V*_*l*_ is made inactive by setting *a*_*l*_ : = 1. Obviously, if later in the search a vertex is moved to *V*_*e*_, then *V*_*l*_ can be selected as a new active empty cluster. In this case, the algorithm sets *a*_*l*_ : = 0 and *e* : = *l*.

We now present an iterated tabu search algorithm for the clique partitioning problem. The algorithm iteratively invokes two procedures, TS (Tabu Search) and GSP (Get Start Partition), which are detailed in the forthcoming subsections.

Consider the following ITS.

1. Compute the initial value for the variable *m* denoting the number of clusters (including the empty one).
2. Generate a feasible solution *P* = (*V*_1_,…, *V*_*m*_) to the problem at random (let *V*_*m*_ in *P* be an empty cluster). Set *a*_*k*_ : = 0, *k* = 1,…, *m*, *e* : = *m*, m* : = *m* − 1, P* : = (*V*_1_*,…, *V*_*m*−1_*), *V*_k_* = *V*_*k*_, *k* = 1,…, *m* − 1, and F* : = *F*(*P*).
3. Apply the tabu search procedure TS(*P*, *m*, *e*, P*, F*).
4. Check if a stopping rule is satisfied. If so, then go to (6). Otherwise go to (5).
5. Apply the procedure GSP(*P*, *m*, *e*, *K*, *L*), where *K* and *L* are randomly chosen values for the solution perturbation parameters. Return to (3).
6. Stop with the partition P* = (*V*_1_*,…, *V*_m*_*). The objective function value on P* is equal to F*.

**Figure 1:**
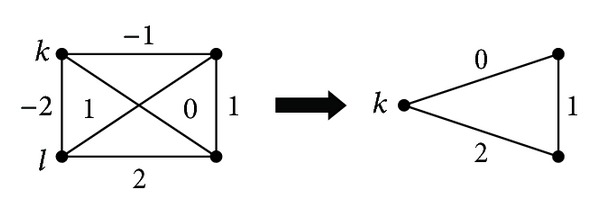
Merging step of the agglomerative heuristic.

A possible option to initialize the variable *m* in Step (1) of ITS is to use a fast constructive heuristic for the CPP. Our choice is to employ a randomized variant of the agglomerative heuristic (AH). This heuristic has been described in [19]. It begins with each vertex declared as a separate cluster and, in each step, merges two clusters into a larger one. This process is modelled by a graph, with vertices representing clusters. In order to select two clusters for agglomeration, AH compares the weights of all the edges of this graph. Among them, an edge having minimum weight is chosen, breaking ties arbitrarily. Let this edge be (*k*, *l*). If its weight is nonnegative, then the algorithm terminates. Otherwise, it merges the clusters represented by vertices *k* and *l* into a single cluster. During this operation, for each cluster *V*_*q*_ with *q* ∉ {*k*, *l*}, the edges (*q*, *k*) and (*q*, *l*) are replaced by one edge connecting *q* and vertex, say *k*, representing the merged cluster. The weight of this edge is set to the sum of the weights of the edges (*q*, *k*) and (*q*, *l*). The merging step of the heuristic is illustrated in Figure 1. The details of AH can be found in [19]. In the ITS framework, we use a version of AH in which an edge for merging is selected randomly from a set of edges having the smallest weights. The size of this set in our implementation is at most 5. Randomization of AH is useful when ITS is run in a multistart fashion.

In Step (2) of the algorithm, a random initial solution to the problem is generated. This is done by first randomly generating a permutation of vertices. Then, a partition is constructed by assigning either ⌊*n*/(*m* − 1)⌋ or ⌈*n*/(*m* − 1)⌉ consecutive vertices from the permutation to each of *m* − 1 nonempty clusters. Before entering the search phase, this partition is saved as the best found solution P*. The number of nonempty clusters in the best solution is denoted by m*. By running the TS procedure, P* is replaced with a solution having a smaller objective function value.

The heart of the ITS algorithm is the loop comprising Steps (3) to (5). Inside this loop, the procedures TS and GSP are executed intermittently. The input to each of them includes the current solution specified by the triplet (*P*, *m*, *e*). The parameters *K* and *L* passed to GSP are used to control the solution perturbation process. The meaning of these parameters is explained later in this section, where also a description of the GSP procedure is given. In Step (4) of ITS, a stopping criterion is required to be specified. It may be any, for example, upper bound on the number of calls to TS or a stopping rule based on the CPU clock. We performed computational experiments using time limit as the stopping condition.

### 2.2. Tabu Search

**Figure 2:**
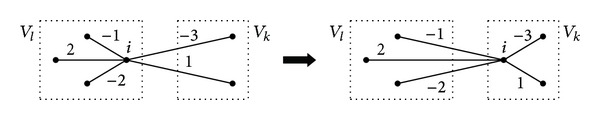
Relocation move.

The type of moves used in our implementation of tabu search is relocation of a vertex from its current cluster to a different one. Given a partition *P* ∈ Π, we define the *relocation neighborhood* *N*_1_(*P*) to be a set of all solutions that can be obtained from *P* by relocating a single vertex. In the search process, it is important to efficiently compute the differences between the values of the objective function at the solutions in the neighborhood *N*_1_(*P*) and the value of the objective function at the current solution *P*. This can be done by taking advantage of an auxiliary *n* × *m* matrix *C* = (*c*_*ik*_), where *c*_*ik*_ = ∑_*j*∈*V*_*k*__ *d*_*ij*_, *i* ∈ *V*, and *k* ∈ {1,…, *m*}. Consider a vertex *i* ∈ *V* and suppose that its owning cluster in *P* is *V*_*l*_. Let Δ(*P*, *i*, *k*) denote the change in the value of the objective function caused by relocating the vertex *i* from the cluster *V*_*l*_ to the cluster *V*_*k*_, *k* ≠ *l* (see Figure 2). In the literature, the cost variation between two solutions, like Δ(*P*, *i*, *k*), is called the move gain. We can express Δ(*P*, *i*, *k*) in terms of the entries of the matrix *C*

(2) $$\begin{array}{c} \Delta \left(P,i,k\right)=c_{ik}-c_{il}. \end{array}$$

In the description of ITS components given below, we will denote by *ρ*(*i*, *P*) the index of the cluster in *P* which the vertex *i* belongs to. Thus, if *i* ∈ *V*_*l*_ ∈ *P*, then *ρ*(*i*, *P*) = *l*. The first component we present is the tabu search procedure. It maintains three data structures to store the tabu status of moves: the matrix *T* = (*t*_*ik*_) with rows and columns corresponding to vertices and clusters, respectively, and lists *τ* and *τ*′. The lists are used to represent, in a compact form, moves that are forbidden for a certain number of iterations. If, for example, vertex *i* is relocated from cluster *V*_*l*_ of size greater than one to a different cluster, then *t*_*il*_ is set to 1, the vertex *i* is appended to the list *τ*, and the cluster index *l* is appended to the list *τ*′. The reason behind the introduction of the lists *τ* and *τ*′ is to efficiently flip *t*_*ik*_ back from 1 to 0, whenever a specified number of iterations have been executed. This number is called *tabu tenure* and is considered as a parameter, denoted as $\bar{t}$, of the TS procedure. Another parameter of TS is the number of iterations $\bar{\alpha }$. The procedure can be stated as follows.

Consider the following TS(*P*, *m*, *e*, P*, F*).

1. Initialize *t*_*ik*_ for each vertex *i* ∈ *V* and each cluster *V*_*k*_, *k* ∈ {1,…, *m*}, with 0. Set *α* : = 0 and *f* : = *F*(*P*).
2. Increase *α* by 1. Set Δ* : = *∞*, Δ′ : = F* − *f*, and *b* : = 0.
3. Iterating through all vertices *i* ∈ *V* and clusters *V*_*k*_, *k* ∈ {1,…, *m*}, such that *k* ≠ *ρ*(*i*, *P*) and *a*_*k*_ = 0, perform the following steps.
  (3.1) Compute *z* : = Δ(*P*, *i*, *k*) by (2). If *z* < Δ′, then proceed to (3.2). Otherwise, check whether one of the following conditions holds: (i) *t*_*ik*_ = 1; (ii) *b* > 0; (iii) |*V*_*ρ*(*i*,*P*)_ | = 1 and *k* = *e*. If so, then go to (3.4); if not, go to (3.3).
  (3.2) Increase *b* by 1 and set Δ* : = *z*, *v* : = *i*, and *q* : = *k* with probability 1/*b*. Go to (3.4).
  (3.3) If *z* < Δ*, then set Δ* : = *z*, *v* : = *i*, *q* : = *k*, and *η* : = 1. Otherwise, if *z* = Δ*, then increase *η* by 1 and set *v* : = *i* and *q* : = *k* with probability 1/*η*.
  (3.4) Repeat (3.1)–(3.3) until all pairs *i*, *k* have been examined.
4. Save *ρ*(*v*, *P*) as *l* and |*V*_*l*_| as *h*. For each *i* ∈ *V*∖{*v*}, subtract *d*_*iv*_ from *c*_*il*_ and add it to *c*_*iq*_. Move the vertex *v* from the cluster *V*_*l*_ to the cluster *V*_*q*_. Increase *f* by Δ*. If *V*_*l*_ becomes emptied, then mark this cluster as inactive by setting *a*_*l*_ : = 1. Otherwise, if *q* = *e*, then perform the following operations. If there exists a cluster *V*_*r*_ such that *a*_*r*_ = 1, then set *a*_*r*_ : = 0 and *e* : = *r*. Otherwise, all clusters appear to be active, and, in this case, increment *m* by 1, add an empty cluster *V*_*m*_ to *P*, initialize the *m*th column of both *T* and *C* with zero vector, and set *e* : = *m*, *a*_*m*_ : = 0.
5. If *b* > 0, then proceed to (6). Otherwise, go to (7).
6. Call the local search procedure LS(*P*, *m*, *e*). Let *P* also denote the solution returned by it. Form a partition $P^{*}:=(V_{1}^{*},\ldots ,V_{\tilde{m}}^{*})$ by identifying nonempty clusters *V*_r_*, $r=1,\ldots ,\tilde{m}$, among *V*_1_,…, *V*_*m*_. Set $m^{*}:=\tilde{m}$, F* : = *F*(P*), and *f* : = F*.
7. If $\alpha =\bar{\alpha }$, then return. Otherwise, perform the following operations. If $\alpha >\bar{t}$, then set *t*_*τ*(1)*τ*′(1)_ : = 0 and remove *τ*(1) and *τ*′(1) from the lists *τ* and *τ*′, respectively. If $\alpha ⩽\bar{t}$, then bypass these modifications of *T*, *τ*, and *τ*′. In both cases, add the vertex *v* at the end of the list *τ*. Check whether *h* = 1. If so, then add the empty cluster index *e* at the end of the list *τ*′ and set *t*_*ve*_ : = 1. If not, then add *l* at the end of *τ*′ and set *t*_*vl*_ : = 1. Go to (2).

In the above description, *α* is the iteration counter, *f* is the value of the current solution, and *b* stands for the number of solutions found in the current iteration, which are better than the best partition, P*, recorded so far. The counter *b* is increased if and only if the move gain Δ is strictly less than the threshold Δ′. In Step (3) of TS, there are two possible cases to consider for a feasible pair consisting of vertex *i* and cluster *V*_*k*_. If Δ < Δ′, then a new improving solution specified by the pair (*i*, *k*) is found. It is always accepted if *b* = 1 and accepted with probability 1/*b* if *b* > 1. If, however, Δ⩾Δ′, then conditions (i)–(iii) in Step (3.1) are checked. Condition (iii) is reasonable because it makes no sense to relocate the last vertex of a cluster to the empty cluster. If at least one of the conditions (i)–(iii) is satisfied, then the pair (*i*, *k*) is immediately rejected. Otherwise, (*i*, *k*) is compared with the best-gain move in Step (3.3). The selected move is represented by the pair (*v*, *q*). In Step (4) of TS, *ρ*(*v*, *P*) and |*V*_*ρ*(*v*,*P*)_| are saved in order to be used later when updating the tabu data structures at the end of the iteration. The same step also updates both the current solution and the matrix *C* and, if needed, introduces a new active empty cluster. After these rearrangements, the local search procedure is applied only when an improving solution was found. The resulting partition is saved as the new best solution. While doing this, the partition is shrunk by removing all empty clusters. Step (7) of TS updates the tabu information. If $\alpha >\bar{t}$, then the tabu status of the oldest pair (vertex, cluster) is revoked by appropriately modifying the data structures *T*, *τ*, and *τ*′. For any *α*, the pair consisting of the selected vertex and, depending on the value of *h*, either its previous cluster or the empty one is made forbidden for the next $\bar{t}$ iterations.

**Figure 3:**
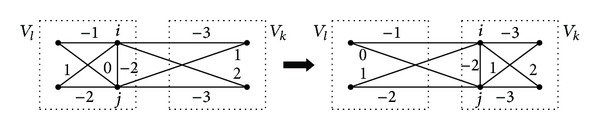
Simultaneously relocating two vertices.

Our local search procedure for the CPP involves moves of two types. One of them is the same as that used in the TS algorithm. Another type of move is to simultaneously relocate two vertices from their current cluster to a different one. Given a partition *P*, two vertices *i* and *j* such that *ρ*(*i*, *P*) = *ρ*(*j*, *P*), and a cluster *k* ≠ *ρ*(*i*, *P*), we denote by *P*(*i*, *j*, *k*) the solution that is derived from *P* by moving vertices *i* and *j* from the cluster *V*_*ρ*(*i*,*P*)_ to the cluster *V*_*k*_. Let *N*_2_(*P*) stand for the set of all solutions that can be obtained in this way. The gain *δ*(*P*, *i*, *j*, *k*) of moving from solution *P* to solution *P*(*i*, *j*, *k*) ∈ *N*_2_(*P*) can be efficiently calculated as follows:

(3) δ(P,i,j,k)=F(P(i,j,k))−F(P)=cik−cil+cjk−cjl+2dij,

where *l* = *ρ*(*i*, *P*) = *ρ*(*j*, *P*). A move of the second type is illustrated in Figure 3. Notice there that relocating a single vertex, *i* or *j*, does not lead to an improvement.

At each iteration, the local search (LS) procedure first explores the neighborhood *N*_2_(*P*) and, if no solution better than *P* is found, then explores the neighborhood *N*_1_(*P*). The procedure consists of the following steps.

Consider the following LS(*P*, *m*, *e*).

1. Randomly generate a permutation of vertices, denoted by (*π*(1),…, *π*(*n*)), and a permutation of clusters, denoted by (*π*′(1),…, *π*′(*m*)).
2. Initialize Δ* with 0.
3. For *i*′ = 1,…, *n* − 1 and *j*′ = *i*′ + 1,…, *n*, do the following.
  (3.1) Set *i* : = *π*(*i*′) and *j* : = *π*(*j*′). If either *ρ*(*j*, *P*) ≠ *ρ*(*i*, *P*) or *d*_*ij*_⩾0, then go to (3.4). Otherwise proceed to (3.2).
  (3.2) Iterating through all clusters *k* : = *π*′(*k*′), *k*′ = 1,…, *m*, such that *k* ≠ *ρ*(*i*, *P*) and *a*_*k*_ = 0, perform the following steps.
    (3.2.1) Compute *z* : = *δ*(*P*, *i*, *j*, *k*) by (3).
    (3.2.2) Check whether *z* < Δ*. If so, then set Δ* : = *z* and *q* : = *k*.
  (3.3) If Δ* < 0, then set *v* : = *i*, *u* : = *j* and go to (7).
  (3.4) Repeat (3.1)–(3.3) until all pairs *i*′, *j*′have been examined.
4. For *i*′ = 1,…, *n*, do the following.
  (4.1) Set *i* : = *π*(*i*′).
  (4.2) Iterating through all clusters *k* : = *π*′(*k*′), *k*′ = 1,…, *m*, such that *k* ≠ *ρ*(*i*, *P*) and *a*_*k*_ = 0, perform the following steps.
    (4.2.1) Compute *z* : = Δ(*P*, *i*, *k*) by (2).
    (4.2.2) Check whether *z* < Δ*. If so, then set Δ* : = *z* and *q* : = *k*.
  (4.3) If Δ* < 0, then set *v* : = *i* and go to (6).
5. Return (because Δ* = 0, which means that no improving move is detected).
6. Update the current solution *P* and auxiliary data as in Step (4) of TS. Go to (8).
7. Let *l* : = *ρ*(*v*, *P*). For each *i* = 1,…, *n* different from *v* and *u*, subtract *d*_*iv*_ + *d*_*iu*_ from *c*_*il*_ and add it to *c*_*iq*_. Also, subtract *d*_*vu*_ from both *c*_*vl*_ and *c*_*ul*_ and add *d*_*vu*_ to both *c*_*vq*_ and *c*_*uq*_. Move the vertices *v* and *u* from the cluster *V*_*l*_ to the cluster *V*_*q*_. If *V*_*l*_ becomes emptied, then set *a*_*l*_ : = 1. Otherwise, if *q* = *e*, then perform the following operations. If there exists a cluster *V*_*r*_ such that *a*_*r*_ = 1, then set *a*_*r*_ : = 0 and *e* : = *r*. If no such cluster exists, then increment *m* by 1, add an empty cluster *V*_*m*_ to *P*, initialize the *m*th column of *C* with zero vector, and set *e* : = *m*, *a*_*m*_ : = 0.
8. If *m* has been increased (either in Step (6) or in Step (7)), then expand *π*′ by setting *π*′(*m*): = *m*. Go to (2).

Each iteration of the described procedure scans the neighborhood *N*_2_(*P*) in Step (3) and the neighborhood *N*_1_(*P*) in Step (4). Both vertices and clusters are considered in the order given by random permutations. Such an approach allows us to introduce some extra randomization in the ITS algorithm. In Step (3) of the LS procedure, only pairs of vertices connected by a negative edge and, of course, belonging to the same cluster are examined. Indeed, if *d*_*ij*_⩾0 for some *i*, *j* ∈ *V*, then, as it follows from (3), it is meaningful to ignore simultaneous relocation of the vertices *i* and *j* and to evaluate moves involving only one of these vertices. For a pair of vertices passing the test in Step (3.1), the aim is to identify a move with negative value of *δ* calculated from (3). If two or more such moves exist, then the one with the minimum *δ* is selected. Throughout this process, the value of the best move is saved as Δ*. The loop in Step (3.2) evaluates the quality of partitions obtained from *P* by relocating the vertices *i* and *j* to other clusters than their own. Provided that Δ* is negative, the index of the best cluster is stored in the variable *q*. If at the end of the loop Δ* < 0, then the current solution *P* is replaced by the partition *P*(*i*, *j*, *q*) ∈ *N*_2_(*P*) in Step (7) of LS. If, however, no improving neighbor in *N*_2_(*P*) is found, then Step (4) is executed. Its structure is very similar to that of Step (3). If, for a vertex *i* and at least one cluster, the value of Δ is negative, then the move involving *i* is accepted and the current solution is replaced with a better one in Step (6). Reaching Step (5) means that a locally optimal solution is obtained, and no improving move of two considered types is available. Step (7) of LS is similar to Step (4) of TS. Little difference is seen in formulas used for updating the matrix *C*.

### 2.3. Solution Perturbation

Another crucial component of the iterated tabu search algorithm is the solution perturbation procedure GSP. When applied within the ITS framework, it produces starting partitions for tabu search. Such partitions are generated by making a certain number of moves. In contrast to the commonly used method when moves are selected randomly, our procedure favours moves that minimize, to some degree, the degradation of the objective function value of the problem. Still, the procedure incorporates a randomization element. At each step, it selects a move at random from a list of the most attractive candidates. The upper limit on the cardinality of this list, denoted by *L*, is a parameter of the procedure. Another parameter, *K*, is the number of vertices that have to be moved from their current clusters to different ones. The input to GSP, of course, includes a partition *P*. In our implementation, ITS submits to GSP the partition that has been returned by the most recent invocation of the TS procedure. While selecting a perturbation move, GSP partially explores the neighborhood *N*_2_(*P*). Basically, GSP is reminiscent of the above described LS algorithm with Steps (4) and (6) removed. The solution perturbation procedure can be described as follows.

Consider the following GSP(*P*, *m*, *e*, *K*, *L*).

1. Set *U* : = *∅* and *W* : = *∅*.
2. Iterating through all vertex pairs *i*, *j* ∈ *V*∖*U* and clusters *V*_*k*_ such that *e* ≠ *k* ≠ *ρ*(*i*, *P*) = *ρ*(*j*, *P*), *a*_*k*_ = 0, and *d*_*ij*_ < 0, perform the following steps.
  (2.1) Compute *z* : = *δ*(*P*, *i*, *j*, *k*) by (3). If |*W* | <*L*, then go to (2.3). Otherwise, proceed to (2.2).
  (2.2) Identify a triplet *w*′ ∈ *W* such that *Z*(*w*′)⩾*Z*(*w*) for all *w* ∈ *W*. If *z* < *Z*(*w*′), then remove *w*′ from *W* and go to (2.3). Otherwise, repeat from (2.1) until all proper combinations of the vertex pair and cluster have been examined.
  (2.3) Create a triplet *w*′′ = (*i*, *j*, *k*) with weight *Z*(*w*′′) = *z* attached to it. Add *w*′′ to *W*.
3. If *W* is empty, then return with *P*. Otherwise, select a triplet *w*, say *w* = (*v*, *u*, *q*), from *W* at random. Add the vertices *v* and *u* to *U*.
4. Update the current solution *P* and auxiliary data as in Step (7) of LS. If |*U* | <*K*, then make the set *W* empty and go to (2). Otherwise, return with the partition *P*.

As can be seen from the description, a single iteration of GSP comprises Steps (2) to (4). When *K* is sufficiently less than *n*, GSP performs ⌈*K*/2⌉ iterations. In each of them, the set *U* of the relocated vertices is enlarged by the addition of a pair of vertices selected in Step (3). In the procedure, *W* stands for the candidate list of the best moves. A move in *W* is represented by the triplet consisting of two vertices and index of their target cluster. In Step (2), the neighborhood *N*_2_(*P*) is searched only partially. Relocating a vertex twice during the run of GSP is prevented by ignoring vertices that belong to the set *U*. Also, like in LS, the search is restricted only to pairs of vertices that are connected by a negative edge. In addition, the use of an empty target cluster is forbidden. The fitness of legal moves (triplets) is evaluated by (3). If the new triplet is better than the worst triplet in the candidate list *W* and the size of *W* is equal to *L*, then the worst triplet in *W* is replaced by the new one. In Step (3), the move to be performed is selected from the list *W* at random. The moved vertices are added to the set *U*. The solution *P* is updated precisely in the same way as in Step (7) of LS. The above outlined process is repeated until the cardinality of the set *U* reaches the prescribed limit *K*.

The value of the parameter *K* for each run of GSP is generated using three secondary parameters *K*_1_, *K*_2_, and *K*_min⁡_. First, an integer *K*′ from the interval [⌊*K*_1_ *n*⌋, ⌈*K*_2_ *n*⌉] is chosen uniformly at random. Then, *K*′ is compared with *K*_min⁡_. If *K*′ > *K*_min⁡_, then *K* is uniformly and randomly chosen from the integers in the interval [*K*_min⁡_, *K*′]. Otherwise, *K* is set to *K*′. Thus, in the general case, *K* is drawn from the interval whose right endpoint is not fixed throughout the execution of the ITS algorithm. The value of the parameter *L* for GSP is an integer randomly drawn from the interval [*L*_1_, *L*_2_], where *L*_1_ and *L*_2_ > *L*_1_ are some constants of the algorithm. The most appropriate values of *K*_1_, *K*_2_, *K*_min⁡_, *L*_1_, and *L*_2_ should be selected experimentally.

## 3. Computational Experiments

The main purpose of experimentation was to show the attractiveness and competitiveness of our approach. In order to evaluate the performance of the developed algorithm, we compared it with two state-of-the-art techniques for the CPP, proposed by Brusco and Köhn [6], namely, the neighborhood search heuristic coupled with the relocation procedure in one case and with the tabu search algorithm in another case. In the study of Brusco and Köhn [6], these algorithms are denoted as NS-R and NS-TS, respectively. When referring to them in this paper, we will use the same names. To be more focused, we do not experiment with other existing methods for the CPP, which are less successful in comparison with both NS-R and NS-TS.

### 3.1. Experimental Protocol

The described algorithm has been coded in the C programming language. Fortran implementations of NS-R and NS-TS were obtained from Brusco and Köhn [6]. All the tests have been carried out on a PC with an Intel Core 2 Duo CPU running at 3.0 GHz. As a testbed for evaluating the performance of the algorithms, we used a set of CPP instances from the literature as well as two sets of additional instances of our own. The first set consists of 7 benchmark instances originally considered by Charon and Hudry [27] (*rand100-100*, *rand300-100*, *rand500-100*, *rand300-5*, *zahn300*, *sym300-50*, and *regnier300-50*) and 6 instances introduced by Brusco and Köhn [6] (*rand200-100*, *rand400-100*, *rand100-5*, *rand200-5*, *rand400-5*, and *rand500-5*). For descriptions of these problem instances, see [6, 27]. In order to test the algorithms more thoroughly, we generated two additional sets of large random CPP instances. The first of them consists of 20 weighted graphs of order 500. The weights of edges are integer numbers drawn uniformly at random from the interval [−5,5] for the first 10 graphs and from the interval [−100,100] for the remaining 10 graphs. The second additional set consists of three subsets, each of cardinality 5. The order of the graphs in the first to third subsets is 1000, 1500, and 2000, respectively. The edge weights are random integers uniformly distributed in range [−100,100].

The TS and GSP procedures described in the previous section have several parameters that affect the performance of the ITS algorithm. Their values were determined by conducting a preliminary experiment. Based on the obtained results, in the main experiments, the TS parameter $\bar{\alpha }$ was fixed at 200. As for the tabu tenure, we have found that a good choice is to take $\bar{t}=\min(10,n/4)$. The parameters used to generate *K* and *L* values were set as follows: *K*_1_ = 0.1, *K*_2_ = 0.6, *K*_min⁡_ = 10, *L*_1_ = 10, and *L*_2_ = 300. The only algorithm's parameter whose value is required to be submitted to our ITS code is a maximum CPU time limit per run.

Given its stochastic nature, we executed the ITS algorithm 10 times on each of the test problems. However, unlike ITS, both NS-R and NS-TS were run only once on each instance of size 500 or less. The reason behind such a decision was our intention to use the original executable code from [6], which does not have an option of restarting the algorithms. For graphs of order greater than 500, we used a very slightly modified version of the source (Fortran) code developed in [6]. In fact, we made just a couple of minor adjustments. First, we increased the size of arrays and matrices from 500 and 500 × 500 to 2000 and 2000 × 2000, respectively. Second, we included in the possibility to restart the algorithms an arbitrary number of times. We capitalized on this possibility in the experiment on the third dataset. Like in the case of ITS, we ran both NS-R and NS-TS 10 times on each instance in this set. It should be noted, however, that, due to a different Fortran compiler we used, the generated executable code may appear to be less efficient than that provided by Brusco and Köhn [6].

As mentioned above, the input to our ITS code includes the time limit per run. We imposed time limits of 100 seconds, 1000 seconds, 2000 seconds, 1 hour, 2 hours, and 5 hours for test problems with *n* ⩽ 200, *n* = 300, 400 ⩽ *n* ⩽ 500, *n* = 1000, *n* = 1500, and *n* = 2000, respectively. The same cutoff times were used for NS-R and NS-TS as well.

### 3.2. Numerical Results

The results of solving CPP instances in the first dataset are summarized in Table 1. Its first column shows the instance names in which the integer preceding “-” indicates the number of vertices. The second column presents the best known values reported in the literature. For *rand100-100*, *rand300-5*, *rand300-100*, *sym300-50*, *regnier300-50*, *zahn300*, and *rand500-100*, the best solutions were obtained by Charon and Hudry [27]. The authors mention that the experiments on these instances took up to several days of the CPU time. For the remaining instances in Table 1, the best known results were reported by Brusco and Köhn [6]. The third column shows the gap of the value of the best solution out of 10 runs (the gap of the average value of 10 solutions) found by ITS to the value displayed in the second column. The fourth column gives the success rate of reaching the best known value $\hat{F}$. The last two columns provide the deviation of the value of the solution delivered by NS-R and, respectively, NS-TS from the value $\hat{F}$. The bottom row shows the results averaged over the whole set of instances.

From Table 1, we see that ITS is superior to both NS algorithms. To be fair, we have to keep in mind that both NS-R and NS-TS were run only once, as opposed to 10 runs in the case of ITS. Therefore, we should compare the results displayed in the last two columns with those shown in parentheses for the ITS algorithm. It can be observed that both variations of NS failed to solve some of the instances of size up to 300. Meanwhile, ITS was able to reach the best known solutions for all of them in each of 10 runs.

In Table 2, we summarize the results of an empirical evaluation of tested algorithms for instances in the second dataset. The structure of this table is the same as that of Table 1. As a basis for comparison, we use, for each instance, the value of the best solution obtained from 10 runs of the ITS algorithm. This value, denoted by $\hat{F}$, is given in the second column. The results from Table 2 indicate that ITS significantly outperforms both reference algorithms in terms of solution quality. A direct comparison of algorithms across problem instances, using $F_{\text{aver}}-\hat{F}$ as a metric for ITS effectiveness, shows that ITS yields better solutions than NS-R and NS-TS in 18 and, respectively, 17 cases out of 20. We see that NS-TS was able to reach the best results for 3 instances in the dataset. Another NS variation, NS-R, has obtained the best solution value in one case only.

Table 3 reports comparative results of ITS with NS-R and NS-TS for instances of size ranging from 1000 to 2000 vertices. The number of vertices is encoded in the instance name. The first three columns of the table have the same meaning as in Table 2. The fourth column displays the gap of the value of the best solution out of 10 runs (the gap of the average value of 10 solutions) found by NS-R to the best value $\hat{F}$. The last column provides these statistics for the NS-TS method. The values shown in the second column were obtained by the ITS algorithm. From the results in Table 3, we see that the superiority of the proposed algorithm over NS-R and NS-TS is more pronounced than in the previous experiments. In fact, ITS dominates both NS-R and NS-TS on all instances in the third dataset. Both the NS variations failed to reach the best value in all cases. By analyzing the results in Tables 2 and 3, we also find that NS-R and NS-TS perform comparably in terms of solution quality, with NS-TS having a slight edge. A similar conclusion has been reached in the study of Brusco and Köhn [6].

## 4. Conclusions

In this paper we have presented an iterated tabu search algorithm for the clique partitioning problem. The described method incorporates tabu search, local search, and solution perturbation procedures. The latter is an essential component of the approach, because, according to our experience, to be successful, a tabu search-based algorithm for graph partitioning type problems should use a sufficiently powerful search diversification mechanism.

Experimental evaluations on three sets of CPP instances of size up to 2000 show that the proposed algorithm is able to produce solutions of high quality. In particular, we can conclude that our algorithm exhibits superior performance compared to the method of Brusco and Köhn. However, we surmise that, for the largest instances in the test suite, the best solutions obtained probably are not the best possible. We have experienced that in order to find improved solutions using ITS a big amount of CPU time is needed. The development of fast and powerful heuristic algorithms for the CPP is an important line of further research.

## Figures and Tables

**Table 1:** Comparison of ITS with NS-R and NS-TS on the CPP instances considered by Brusco and Köhn.

Instance	Best known	ITS		NS-R	NS-TS
	Value $\hat{F}$	$F_{\text{best}}-\hat{F}$ ($F_{\text{aver}}-\hat{F}$)	Succ.	$F-\hat{F}$	$F-\hat{F}$
rand100-5	− 1407	0 (0)	10	0	0
rand100-100	− 24296	0 (0)	10	0	0
rand200-5	− 4079	0 (0)	10	0	0
rand200-100	− 74924	0 (0)	10	0	0
rand300-5	− 7732	0 (0)	10	3	3
rand300-100	− 152709	0 (0)	10	0	0
sym300-50	− 17592	0 (0)	10	0	0
regnier300-50	− 32164	0 (0)	10	0	0
zahn300	− 2504	0 (0)	10	1	0
rand400-5	− 12133	0 (0.1)	9	37	13
rand400-100	− 222757	0 (42.0)	7	110	208
rand500-5	− 17127	0 (5.2)	7	58	41
rand500-100	− 309125	118 (224.3)	0	118	735

Average		9.1 (20.9)	8.7	25.2	76.9

**Table 2:** Comparison of ITS with NS-R and NS-TS on the CPP instances of size 500.

Instance	Best value	ITS		NS-R	NS-TS
	$\hat{F}$	$F_{\text{best}}-\hat{F}$ ($F_{\text{aver}}-\hat{F}$)	Succ.	$F-\hat{F}$	$F-\hat{F}$
p500-5-1	− 17691	0 (10.5)	1	30	0
p500-5-2	− 17169	0 (1.5)	6	29	0
p500-5-3	− 16815	0 (1.0)	3	92	129
p500-5-4	− 16808	0 (3.5)	8	71	70
p500-5-5	− 16957	0 (0.0)	10	53	51
p500-5-6	− 16615	0 (2.9)	6	134	77
p500-5-7	− 16649	0 (8.0)	1	59	71
p500-5-8	− 16756	0 (0.9)	7	153	142
p500-5-9	− 16629	0 (5.8)	4	17	77
p500-5-10	− 17360	0 (0.0)	10	60	62
p500-100-1	− 308896	0 (18.9)	4	9	1827
p500-100-2	− 310163	0 (171.5)	2	1713	858
p500-100-3	− 310477	0 (94.7)	4	2149	718
p500-100-4	− 309567	0 (282.9)	1	904	796
p500-100-5	− 309135	0 (41.6)	7	2528	0
p500-100-6	− 310280	0 (66.7)	7	0	722
p500-100-7	− 310063	0 (5.8)	9	1456	2088
p500-100-8	− 303148	0 (344.0)	5	2205	1686
p500-100-9	− 305305	0 (7.2)	9	1232	1110
p500-100-10	− 314864	0 (7.6)	9	76	106

Average		0 (53.7)	5.7	648.5	529.5

**Table 3:** Comparison of ITS with NS-R and NS-TS on larger CPP instances.

Instance	Best value	ITS	NS-R	NS-TS
	$\hat{F}$	$F_{\text{best}}-\hat{F}$ ($F_{\text{aver}}-\hat{F}$)	$F_{\text{best}}-\hat{F}$ ($F_{\text{aver}}-\hat{F}$)	$F_{\text{best}}-\hat{F}$ ($F_{\text{aver}}-\hat{F}$)
p1000-1	− 883359	0 (2190.5)	7099 (11056.4)	6467 (12371.8)
p1000-2	− 879792	0 (1507.5)	7851 (12533.1)	7262 (11585.2)
p1000-3	− 862969	0 (1690.8)	6518 (9638.3)	7516 (9905.1)
p1000-4	− 865754	0 (1167.4)	8388 (12325.2)	7346 (10487.2)
p1000-5	− 887314	0 (2224.1)	6990 (11777.1)	4777 (10263.6)
p1500-1	− 1614791	0 (6883.5)	22179 (26820.6)	17669 (23080.2)
p1500-2	− 1642442	0 (5174.1)	21126 (26973.8)	20750 (27272.0)
p1500-3	− 1600857	0 (2457.3)	12599 (22708.8)	12005 (19924.9)
p1500-4	− 1633081	0 (3884.2)	19236 (25841.2)	12248 (21993.8)
p1500-5	− 1585484	0 (3005.8)	16894 (24918.8)	12430 (20878.4)
p2000-1	− 2489880	0 (4229.5)	32501 (40603.7)	25242 (40091.7)
p2000-2	− 2479127	0 (4504.4)	34047 (41348.2)	21122 (37640.4)
p2000-3	− 2527119	0 (4480.4)	29320 (37828.0)	18350 (33717.8)
p2000-4	− 2511914	0 (4461.9)	28342 (38291.0)	32269 (42376.9)
p2000-5	− 2499690	0 (7846.1)	32928 (42543.9)	30561 (37686.7)

Average		0 (3713.8)	19067.9 (25680.5)	15734.3 (23951.7)
